# Essential Reads in Rhinology: A Bibliometric Analysis

**DOI:** 10.1007/s12070-024-05055-2

**Published:** 2024-09-23

**Authors:** Ariana L. Shaari, Shreya Bhalla, Rebecca Ho, Anup Dupaguntla, Sylvia Zabielski, Wayne Hsueh, Jean Anderson Eloy, Andrey Filimonov

**Affiliations:** 1 Department of Otolaryngology – Head and Neck Surgery, Rutgers New Jersey Medical School, Newark, NJ USA; 2 Rutgers Robert Wood Johnson Medical School, New Brunswick, NJ USA; 3 Center for Skull Base and Pituitary Surgery, Neurological Institute of New Jersey, Rutgers New Jersey Medical School, Newark, NJ USA; 4 Department of Neurological Surgery, Rutgers New Jersey Medical School, Newark, NJ USA; 5 Department of Ophthalmology and Visual Science, Rutgers New Jersey Medical School, Newark, NJ USA; 6 Department of Otolaryngology and Facial Plastic Surgery, Saint Barnabas Medical Center - RWJBarnabas Health, Livingston, NJ USA

**Keywords:** Rhinology, Bibliometric analysis, Gender disparities, Surgical education, Otolaryngology

## Abstract

**Aims:**

Rhinology is a progressive subspecialty within otolaryngology. Bibliometric analysis is a powerful method to survey the landscape of literature on a specific topic and identify publication trends. We aimed to analyze the 50 most impactful English-language rhinology manuscripts of all time to create a targeted reading list for otolaryngologists.

**Material and Methods:**

The Journal Citation Report within the Web of Science database was utilized to identify articles relating to rhinology. Web of Science Research categories were restricted to otolaryngology. The articles were then ranked by number of citations. The top 50 articles of all time ranked by number of citations were analyzed. The articles were analyzed for publication year, journal impact factor, citation density, first author (FA), and senior author (SA) name, country, and institution. Statistical analyses were performed.

**Results:**

Most articles were published in the 2000s (N = 22, 44%) and 1990s (N = 11, 22%). Male FA (N = 37, 74%) and SA (N = 43, 86%) published most articles. Most articles were clinical studies (N = 36, 72%) followed by clinical guidelines/expert opinions. Most articles were published in the United States (N = 28, 56%) and England (N = 9, 18%). There were more female FAs of guidelines/position papers than basic lab, reviews, and clinical studies. England had more female FAs than other countries, and the United States had more female SA. There was an average of 585.2 citations per study.

**Conclusion:**

This investigation highlights the most influential literature within rhinology. The most impactful studies are consensus statements, large multicenter studies, and technique studies. Significant gender disparities in authorship exist.

## Introduction

Rhinology is a rapidly evolving subspecialty in otolaryngology that refers to the study of the nose and paranasal sinuses. The earliest records of the study of the nasal structures date back to ancient periods [1]. Further discoveries into the anatomy of the paranasal sinuses has been attributed to Emil Zuckerkandl in 1870s Austria [1, 2]. His meticulous anatomical investigations laid the foundation for new medical and surgical advances in rhinology, such as the development of the endoscope, nasal speculum, and followed by the nasal endoscope in the late 1870s [3–7]. The twentieth century saw advances in specific procedures such as septoplasties and reduction rhinoplasty [2]. In the mid-1900s the utilization of microscopes in nasal operations and new immunologic insights into the difference between allergic and nonallergic pathologies helped advance knowledge and treatment of nasal pathology [2]. Through the twenty-first century research in rhinology has allowed for advances in the technical, pharmacologic, and digital aspects of rhinology [8].

To this day research and innovation continue to underlie rhinology. With new technical advances and insights into disease processes frequently emerging, it can be challenging to remain up to date on the expanding bodies of knowledge in rhinology [9]. Bibliometric analysis is a powerful tool used to analyze the landscape on specific topics and identify publication trends about specific scholarly topics [10, 11]. Within the otolaryngology literature, several bibliometric analyses have been conducted on topics such as vestibular schwannomas, obstructive sleep apnea, and velopharyngeal insufficiency [12–14]. Such studies that accumulate the most popular articles can help practitioners, trainees, and students gain a deeper understanding of a particular topic.

In this investigation, we set out to determine the most influential literature of all time in rhinology to assemble a modern-day reading list for the otolaryngologist and otolaryngology trainees. We secondarily aimed to evaluate authorship trends to shed light on gender disparities.

## Methods

Using methods similar to other bibliometric analyses, the Journal Citation Report within the Web of Science database was utilized to identify articles relating to rhinology (October 2023, New York, New York) [15, 16]. The Web of Science database was utilized as it is known to be one of the most extensive electronic databases internationally [17]. Additionally, previous work has demonstrated the Web of Science database to be the most comprehensive and reliable search platform for the purposes of bibliometric analysis [18]. All journals were included in the search. No temporal limitations were included. The terms utilized in the search are displayed in the Appendix. Web of Science Research Categories were restricted to otolaryngology.

The articles were then ranked by number of citations (“top articles”). Articles not relating to rhinology were excluded. The top 50 articles of all time were isolated and analyzed. All articles were exported to Microsoft Excel. The following information was extracted from each article: publication title, year of publication, journal, journal impact factor (derived from the Journal Citations Report Database), first author gender, first author’s country of affiliation, senior author gender, and citation density. Citation density was calculated using methods similar to previously published papers [19]. Descriptive and statistical analysis were performed using IBM SPSS.

## Results

The aforementioned search string was inputted into the Journal Citation Report within the Web of Science database and retrieved 119,861 articles. After filtering the Web of Science Category for Otolaryngology, a total of 19,232 articles were retrieved. The top 50 articles, defined as the articles with the most overall citations, were identified. The articles available through the Web of Science database were published between 1940 and 2023.

The details, including year, first author gender, country, and number of citations, on the top 50 most cited articles are provided in Table 1. The top 50 articles were published between 1970 and 2020. Articles had a total of 24,959 citations. The average citation density per study was 44.25. Of the 50 top articles, 26% (N = 13) had a female first author (*p* < 0.001) and 14% (N = 7) had a female senior author (*p* < 0.0001).

**Table 1:** Top 50 articles relating to rhinology in the English literature

Rank	Authors	Article title	Publication year	First author gender	Senior author gender	Country of affiliation	Number of authors	Number of citations
1	Fokkens et al.	European Position Paper on Rhinosinusitis and Nasal Polyps 2012	2012	Female	Male	Netherlands	29	3283
2	Lechien et al.	Olfactory and Gustatory Dysfunctions as a Clinical Presentation of Mild-to-Moderate Forms of the Coronavirus Disease (Covid-19): A Multicenter European Study	2020	Male	Male	France	6	1663
3	Hadad et al.	A Novel Reconstructive Technique After Endoscopic Expanded Endonasal Approaches: Vascular Pedicle Nasoseptal Flap	2006	Male	Male	Argentina	7	1270
4	Hopkins et al.	Psychometric Validity of the 22-Item Sinonasal Outcome Test	2009	Female	Male	England	5	1081
5	Stewart et al.	Development and Validation of the Nasal Obstruction Symptom Evaluation (Nose) Scale	2004	Male	Female	United States	6	774
6	Rosenfeld et al.	Clinical Practice Guideline (Update): Adult Sinusitis	2015	Male	Female	United States	12	761
7	Lund et al.	Staging For Rhinosinusitis	1997	Female	Male	United States	2	733
8	Kobal et al.	Multicenter Investigation of 1036 Subjects Using a Standardized Method for the Assessment of Olfactory Function Combining Tests of Odor Identification, Odor Discrimination, and Olfactory Thresholds	2000	Male	Male	Germany	9	645
9	Lanza et al.	Adult Rhinosinusitis Defined	1997	Male	Male	United States	2	602
10	Chandler et al.	Pathogenesis of Orbital Complications in Acute Sinusitis	1970	Male	Male	United States	3	601
11	Bolger et al.	Paranasal Sinus Bony Anatomic Variations and Mucosal Abnormalities—Ct Analysis for Endoscopic Sinus Surgery	1991	Male	Male	United States	3	598
12	Kennedy et al.	Functional Endoscopic Sinus Surgery—Theory and Diagnostic Evaluation	1985	Male	Male	United States	4	524
13	Moein et al.	Smell Dysfunction: A Biomarker For Covid-19	2020	Female	Male	Iran	6	504
14	Bent et al.	Diagnosis of Allergic Fungal Sinusitis	1994	Male	Male	United States	2	498
15	Hummel et al.	Position Paper on Olfactory Dysfunction	2017	Male	Female	United States	39	488
16	Gliklich et al.	The Health Impact of Chronic Sinusitis in Patients Seeking Otolaryngologic Care	1995	Male	Male	United States	2	480
17	Seidman et al.	Clinical Practice Guideline: Allergic Rhinitis	2015	Male	Female	United States	21	478
18	Vaira et al.	Anosmia and Ageusia: Common Findings in Covid-19 Patients	2020	Male	Male	Italy	4	469
19	Lund et al.	European Position Paper on Endoscopic Management of Tumours of the Nose, Paranasal Sinuses and Skull Base Introduction	2011	Female	Male	England	4	447
20	Stammberger et al.	Functional Endoscopic Sinus Surgery—Concept, Indications and Results of the Messerklinger Technique	1990	Male	Male	Austria	2	431
21	Brämerson et al.	Prevalence of Olfactory Dysfunction:: The Skovde Population-Based Study	2004	Female	Male	Sweden	5	430
22	Turner et al.	Incidence and Survival in Patients with Sinonasal Cancer: A Historical Analysis of Population-Based Data	2012	Male	Male	United States	2	394
23	Temmel et al.	Characteristics of Olfactory Disorders in Relation to Major Causes of Olfactory Loss	2002	Male	Male	Austria	6	359
24	Hopkins et al.	The Lund-Mackay Staging System for Chronic Rhinosinusitis: How is it Used and What Does it Predict?	2007	Female	Male	England	5	346
25	Stewart et al.	Outcomes After Nasal Septoplasty: Results from the Nasal Obstruction Septoplasty Effectiveness (Nose) Study	2004	Male	Male	United States	7	346
26	Hummel et al.	Effects of Olfactory Training in Patients with Olfactory Loss	2009	Male	Male	Germany	6	341
27	Senior et al.	Long-Term Results of Functional Endoscopic Sinus Surgery	1998	Male	Male	United States	6	330
28	Vaira et al.	Objective Evaluation of Anosmia and Ageusia in Covid-19 Patients: Single-Center Experience on 72 Cases	2020	Male	Male	Italy	13	328
29	Yan et al.	Association of Chemosensory Dysfunction and Covid-19 in Patients Presenting with Influenza-Like Symptoms	2020	Female	Male	United States	5	324
30	Lund et al.	Quantification for Staging Sinusitis	1995	Female	Male	England	16	322
31	Hummel et al.	Screening of Olfactory Function with a Four-Minute Odor Identification Test: Reliability, Normative Data, and Investigations in Patients with Olfactory Loss	2001	Male	Male	Germany	4	310
32	Nicolai et al.	Endoscopic Surgery for Malignant Tumors of the Sinonasal Tract and Adjacent Skull Base:: A 10-Year Experience	2008	Male	Male	Italy	8	296
33	Patel et al.	Primary Mucosal Malignant Melanoma of the Head and Neck	2002	Female	Male	United States	10	292
34	Zanation et al.	Nasoseptal Flap Reconstruction of High Flow Intraoperative Cerebral Spinal Fluid Leaks During Endoscopic Skull Base Surgery	2009	Male	Male	United States	7	285
35	Chakrabarti et al.	Fungal Rhinosinusitis: A Categorization and Definitional Schema Addressing Current Controversies	2009	Male	Male	India	21	278
36	Bachert et al.	Nasal Polyposis: From Cytokines to Growth	2000	Male	Male	Belgium	5	267
37	Suzuki et al.	Identification of Viruses in Patients with Postviral Olfactory Dysfunction	2007	Male	Male	Japan	7	258
38	Deconde et al.	Prevalence of Polyp Recurrence After Endoscopic Sinus Surgery for Chronic Rhinosinusitis with Nasal Polyposis	2017	Male	Male	United States	6	257
39	Hanna et al.	Endoscopic Resection of Sinonasal Cancers with and Without Craniotomy Oncologic Results	2009	Male	Male	United States	6	256
40	Wallwork et al.	A Double-Blind, Randomized, Placebo-Controlled Trial of Macrolide in the Treatment of Chronic Rhinosinusitis	2006	Male	Male	Australia	5	246
41	Yan et al.	Self-Reported Olfactory Loss Associates with Outpatient Clinical Course in Covid-19	2020	Female	Male	United States	5	245
42	Smith et al.	Predictive Factors and Outcomes in Endoscopic Sinus Surgery for Chronic Rhinosinusitis	2005	Male	Female	United States	6	245
43	Frasnelli et al.	Olfactory Dysfunction and Daily Life	2005	Male	Male	Germany	2	244
44	Havas et al.	Prevalence of Incidental Abnormalities on Computed Tomographic Scans of the Para-Nasal Sinuses	1988	Male	Male	Australia	3	243
45	May et al.	Complications of Endoscopic Sinus Surgery—Analysis of 2108 Patients—Incidence and Prevention	1994	Male	Male	United States	4	238
46	Kaye et al.	Covid-19 Anosmia Reporting Tool: Initial Findings	2020	Female	Male	United States	6	232
47	Chen et al.	The Epidemiology of Chronic Rhinosinusitis in Canadians	2003	Female	Female	Canada	3	231
48	Ragab et al.	Evaluation of the Medical and Surgical Treatment of Chronic, Rhinosinusitis: A Prospective, Randomised, Controlled Trial	2004	Male	Female	United States	3	230
49	Friedman et al.	Hydroxyapatite Cement .2. Obliteration and Reconstruction of the Cat Frontal-Sinus	1991	Male	Male	England	6	229
50	Féron et al.	New Techniques for Biopsy and Culture of Human Olfactory Epithelial Neurons	1998	Male	Male	United States	4	227

Most studies were clinical studies (N = 36, 72%) followed by clinical guidelines/expert opinion/position papers (N = 9, 18%), reviews (N = 3, 6%) and basic science papers (N = 2, 4%). There were disparities between article type and female first author. Female first and senior authors published significantly more clinical guidelines/expert opinion/position papers than any other types of studies. There were no significant differences between gender of first or senior author and article type (Table 2).

**Table 2:** Number of female first and senior authors by article type

Authorship	Journal	Females (n)	Females (%)	Phi coefficient	*P* value
*First authors*					
	Guidelines/expert opinion/position papers	5	55.56	0.316	0.035
	Clinical	8	22.22	− 0.138	0.333
	Review	0	0	− 0.15	0.514
	Basic science	0	0	− 0.121	0.685
*Senior authors*					
	Guidelines/expert opinion/position papers	3	33.33	0.261	0.082
	Clinical	4	11.11	− 0.134	0.353
	Review	0	0	− 0.102	0.868
	Basic science	0	0	− 0.082	0.950

The *Laryngoscope* published the most articles (N = 15, 30%) followed by *Otolaryngology-Head and Neck Surgery* (N = 10, 20%) and *Archives of Otolaryngology-Head and Neck Surgery* (N = 6, 12%) (Table 3). The average journal impact factor was 3.19. The *International Forum of Allergy and Rhinology* had significantly more female first authors compared to all journals; however, there was no significant difference between any of the journals and the number of female senior authors.

**Table 3:** Number of female first and senior authors by journal

Authorship	Journal	Females (n)	Females (%)	Phi coefficient	*P* value
*First authors*					
	Rhinology	2	66.67	0.234	0.140
	European Archives of Oto-rhino-laryngology	0	0	− 0.175	0.398
	Laryngoscope	2	13.33	− 0.189	0.195
	Clinical Otolaryngology	1	100	0.241	0.187
	Otolaryngology-head and Neck Surgery	3	30	0.046	0.748
	Archives of Otolaryngology-Head and Neck Surgery	0	0	− 0.219	0.253
	International Forum of Allergy and Rhinology	3	100	0.426	0.038
	Head and Neck-journal for the Sciences and Specialties of the Head and Neck	1	33.33	0.042	0.766
	Annals of Otology Rhinology and Laryngology	1	50	0.112	0.450
	American Journal of Rhinology	0	0	− 0.121	0.685
	American Journal of Rhinology and Allergy	0	0	− 0.085	0.950
*Senior authors*					
	Rhinology	1	33.33	0.141	0.345
	European Archives of Oto-rhino-laryngology	0	0	− 0.119	0.728
	Laryngoscope	3	20	0.113	0.429
	Clinical Otolaryngology	0	0	− 0.058	0.705
	Otolaryngology-Head and Neck Surgery	3	30	0.231	0.120
	Archives of Otolaryngology-Head and Neck Surgery	0	0	− 0.149	0.530
	International Forum of Allergy and Rhinology	0	0	− 0.102	0.868
	Head and Neck-journal for the Sciences and Specialties of the Head and Neck	0	0	− 0.102	0.868
	Annals of Otology Rhinology and Laryngology	0	0	− 0.082	0.950
	American Journal of Rhinology	0	0	− 0.082	0.950
	American Journal of Rhinology and Allergy	0	0	− 0.058	0.705

Of the top 50 articles, the United States published the most articles (N = 25, 50%) followed by England (N = 5, 10%) (Table 4). England had significantly more female first authors compared to other countries (*p* = 0.019) (Table 5). The United States had significantly more female senior authors compared to other countries (*p* = 0.036).

**Table 4:** Top countries

Country	Number
United States	25 (50%)
England	5 (10%)
Germany	4 (8%)
Italy	3 (6%)
Austria	2 (4%)
Australia	2 (4%)
Netherlands	1 (2%)
France	1 (2%)
Argentina	1 (2%)
Iran	1 (2%)
Sweden	1 (2%)
India	1 (2%)
Belgium	1 (2%)

**Table 5:** Number of female first and senior authors by country

Authorship	Country	Females (n)	Females (%)	Phi coefficient	*P* value
*First author*					
	England	4	80	0.41	0.019
	United States	5	20	− 0.14	0.337
	Germany	0	0	− 0.17	0.398
*Senior author*					
	England	0	0	− 0.13	0.618
	United States	6	24	0.29	0.036
	Germany	0	0	− 0.12	0.728

Most articles were published in the 2000s (N = 22, 44%) followed by in the 1990s (N = 11, 22%). This decreased during the 2010s (N = 7, 14%) and 2020s (N = 7, 14%). This number decreased in the 2010s (N = 7, 14%) and 2020s (N = 7, 14%). There were an average of 7.2 authors per study (Table 6). There were no significant differences found between the number of female and male first authors, or between female and male senior authors. There were also no significant differences between the number of authors per study and the gender of the first or senior author. There was an average of 499.2 citations per study.

**Table 6:** Mean number of authors per study by female and male first and senior authors

Author type	Females	Males	*P* value
First author	7.77 (2.73–10.96)	7.00 (4.75–9.25)	0.744
Senior author	12.86 (3.88–25.46)	6.28 (4.75–7.81)	0.237

**Fig. 1:**
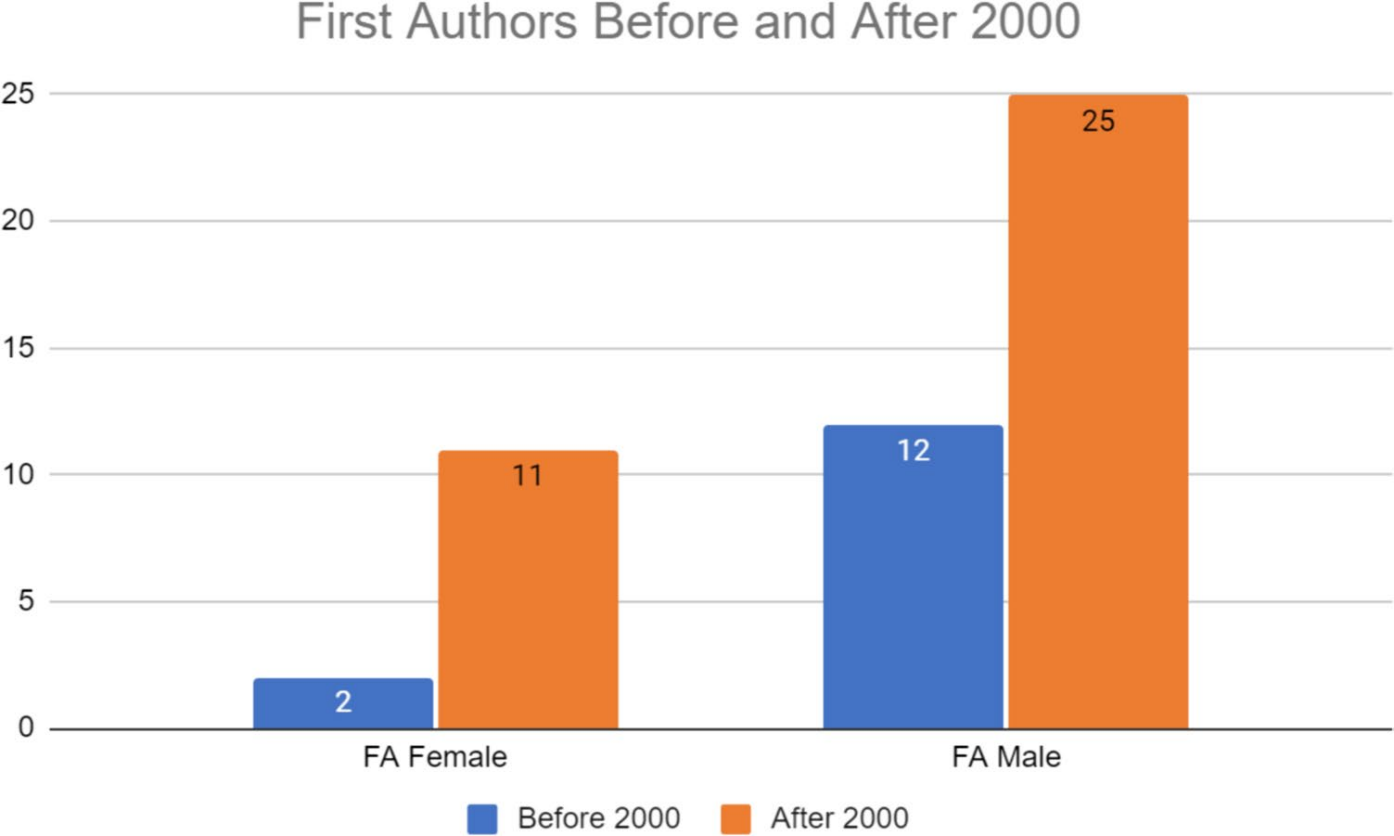
Number of female, male, and total first authors (FA) before and after 2000

**Fig. 2:**
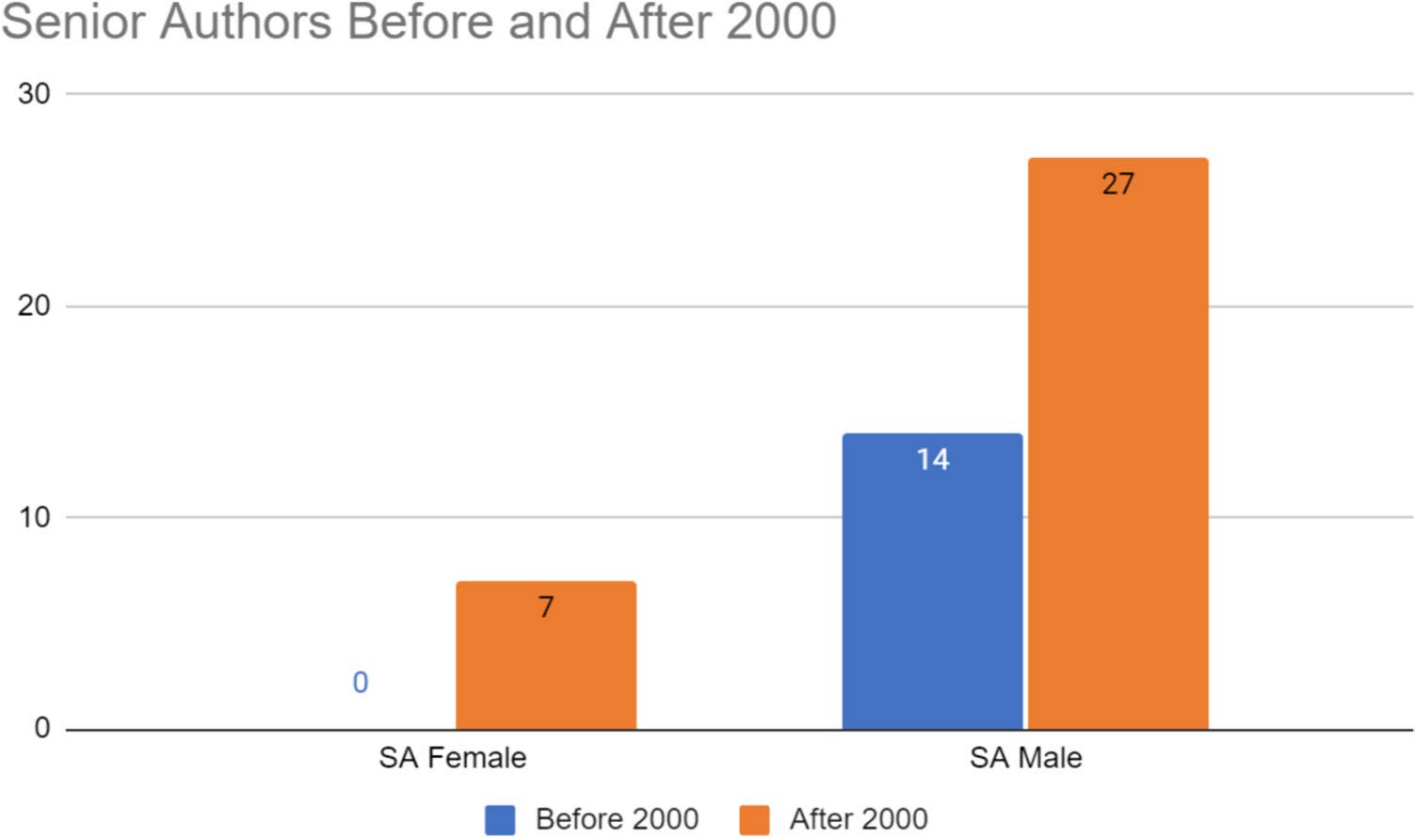
Number of female, male, and total senior authors (SA) before and after 2000

**Fig. 3:**
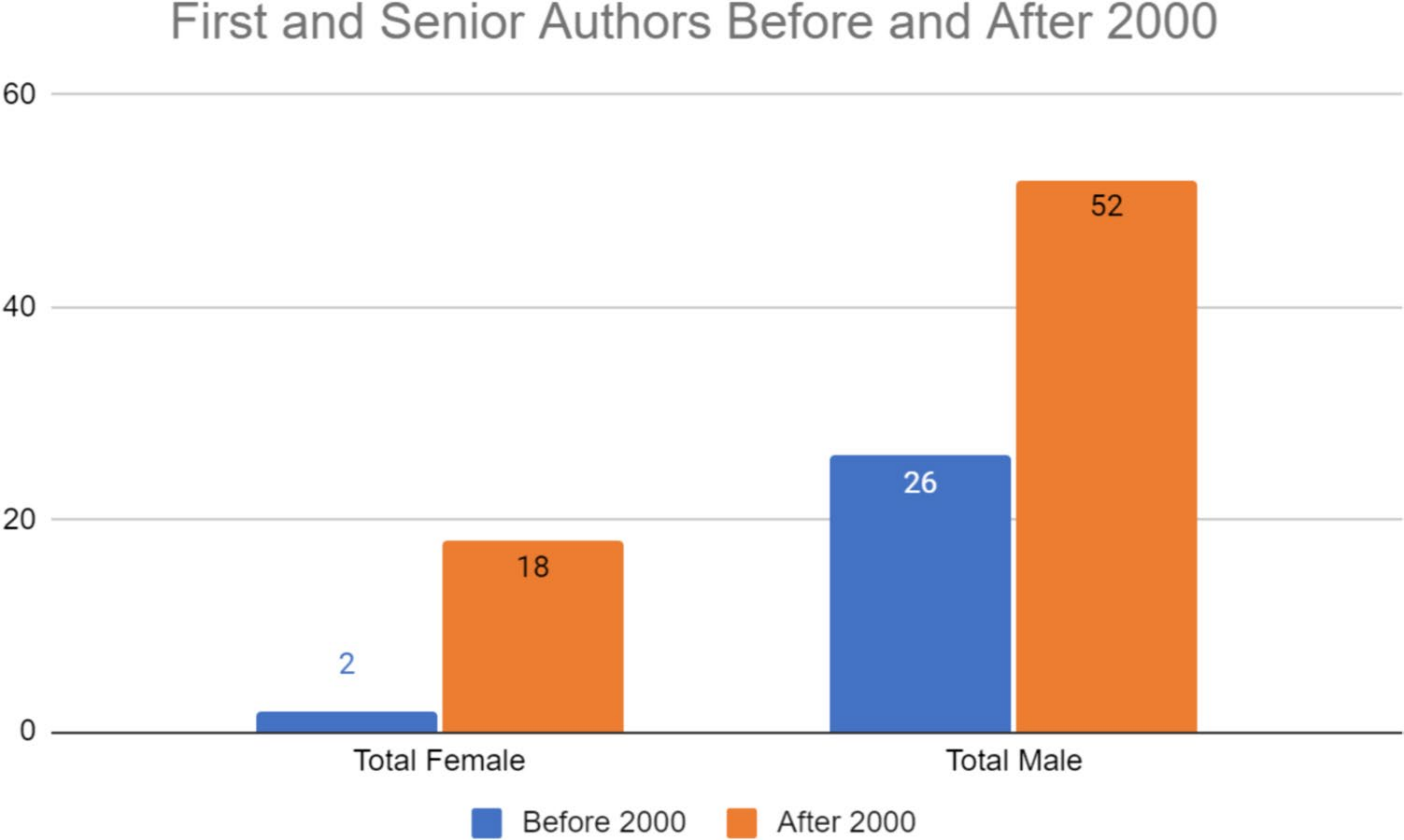
Number of female, male, and total authors before and after 2000

Gender disparities exist in both senior and first authors before and after 2000. The number of female authors increased following 2000 (N = 2 vs. N = 18). The number of male authors also increased after 2000 (N = 26 vs. N = 52) (Figs. 1, 2 and 3). Figure 1 reveals that before 2000 there were more male first authors than female first authors. After 2000, there was a rise in the number of female first authors although that number still lagged behind that of male first authors. Figure 2 demonstrates gender disparities in senior authors before and after 2000. Of note, there were no female senior authors before 2000 but this number jumped after 2000 (N = 0 and N = 14, respectively). After 2000, there were still nearly double as male senior authors compared to females. Overall, females comprised 14% of total first authors before 2000; after 2000, females comprised 32%. Regardless of the year, males comprised the majority of first and senior authorship.

## Discussion

This investigation analyzed the top 50 most cited articles of all time in rhinology and evaluated trends in authorship with a particular emphasis on gender disparities. Overall, our findings were consistent with the literature describing a disparity in the rhinology workforce. The present study serves as the first cumulative review of the most impactful literature in rhinology.

Overall, the *Laryngoscope* published the most articles (N = 15, 30%) followed by *Otolaryngology-Head and Neck Surgery* (N = 10, 20%) and *Archives of Otolaryngology-Head and Neck Surgery* (N = 6, 12%). The most cited article was the “European Position Paper on Rhinosinusitis and Nasal Polyps'' published by Fokkens et al. [20], containing 3283 citations. The article is a summary of definitions and classifications, as well as epidemiology, differentials, and management of rhinosinusitis polyps [20]. It was particularly noteworthy for its discussions of variations in the pathophysiology and treatment of chronic rhinosinusitis with and without nasal polyps.

The oldest article on the list is “The Pathogenesis of Orbital Complications in Acute Sinusitis” published in 1970 by Chandler et al. in *The Laryngoscope* [21]. This article, deemed a “classic” article of rhinology, set a framework for the presentation, pathogenesis, classification, and treatment of infections of the orbit following sinusitis [21, 22]. Although some principles discussed in the original manuscript have changed since its inception, many aspects involving the classification and treatment remain relevant today [22].

Our findings were both consistent and in contrast to literature describing trends in rhinology authorship. We found that overall 26% of first authors (N = 13, 26%) and 14% of senior authors (N = 7, 14%) were female, highlighting a stark disparity in authorship. A 2021 study by Halderman et al. analyzing authorship of allergy and rhinology publications between 2008 and 2018 in 4 journals, found that females composed 23% of authors overall and female first authorship increased significantly, but there was no change in female senior authorship [23]. Despite this increase and an increase in the overall number of women in medicine, there has not been a proportional increase in the number of papers published by female authors [24]. Our findings were consistent with the literature regarding a disparity in authorship; however, our findings captured data outside of the 2018 range as reported by Halderman et al. publishing in high-impact journals is an essential component of academia. Women’s underrepresentation in high-impact journals may have an impact on the representation and promotion of female faculty within otolaryngology [25]. Identifying these trends in female authorship may help shed light on the current and future directions of females in academic rhinology.

Previous studies on female representation in otolaryngology leadership demonstrated that women hold only 18% of directorship positions and 5% of chair positions [26]. This disparity extends into fellowship leadership. Females comprise only 5.7% of rhinology fellowship director positions. Despite this, female fellowship directors have been found to have higher research productivity than males [27]. Other studies suggest that a lack of women in leadership is a key reason for an overall lack of publications from women compared to men, even though there is an increasing number of female physicians [24]. The lack of females in leadership positions suggests the need for female-oriented mentorship programs, which have been shown to promote and retain females in academic medicine [28]. Female tailored mentorship programs have been demonstrated to be associated with an increase in productivity and promotion of medical faculty, which in turn could increase the number of women in leadership positions within rhinology.

Currently, no published literature has analyzed the top rhinology papers for gender disparities in authorship. The current study found notable disparities in authorship regarding first author gender and publication type. Out of the 50 articles examined, there were significantly more female first authors who published guidelines/position papers/expert opinions compared to other article types (clinical studies, reviews, basic science studies). The authors of position papers and clinical practice guidelines are often front runners in the field who are typically selected by invitation. A year-long study of gender differences in the authorship of Canadian and North American published otolaryngology clinical practice guidelines over 17 years found that females accounted for 21.2% of authors overall [29]. Moreover, they found that female otolaryngologist representation was highest in rhinology (28.3%); however, they found that there were no gender differences across first or senior authors and by subspecialty [29]. Although only 5.7% of rhinology fellowship leaders are female, females have been found to have higher research productivity than males [27].

A 2022 study that analyzed the top 3 medical journals, found that female scientists published fewer clinical trials across all fields, which is mirrored in our results regarding otolaryngology [30]. It has been suggested that this difference may be due to differences in training and career decisions between genders [30]. Additionally, previous studies have demonstrated that at senior levels, the research productivity of female otolaryngologists equals or exceeds that of males [31]. Our results suggest that despite the increase in female first authorship during the time studied, female leaders remain underrepresented in rhinology.

This bibliometric analysis includes the top 50 publications between 1970 and 2023. We found that after 2000, the number of female-authored studies increased over time; however, authorship disparities still existed over time. This trend has been seen in both radiology and ophthalmology where female authorship has been increasing over the past decade, but remains underrepresented [32, 33]. Additionally, a Canadian study from 2023, found that female otolaryngologists had equal research productivity compared to their male counterparts, but remained underrepresented in publications [34]. It can be hypothesized that these disparities may exist across disciplines due to bias from the journals themselves or simply a lower number of papers being submitted for review from female first authors. In contrast, other studies have shown that women have lower academic productivity compared to men in plastic surgery in junior positions [35]. Productivity plays a significant role in grant funding as well as academic promotion meaning that men receive the majority of grant funding and can submit more papers for publication than women [35]. Gender disparities are prevalent in society in many other fields in addition to medicine, which can hinder female professional development and undervalue the contributions of females [36].

Most of the top articles were published in the United States (N = 25, 50%) followed by England (N = 5, 10%). These results support previous research in other disciplines such as stem cell transplantation and oncology which demonstrated that high-income countries, such as the United States, had more publications than low-income countries [37, 38]. It is possible that high-income countries are able to produce more publications due to increased research capabilities, especially in research that requires highly specialized and expensive equipment. These fiscal limitations as well as other cultural and social norms could potentially be responsible for the global disparities seen in top publications.

Additionally, England had significantly more female first authors compared to other countries. The United States and Germany had significantly fewer female first authors compared to other countries depicted in Table 6. The United States had significantly more female senior authors compared to other countries (*p* = 0.04). A 2023 German study found that female otolaryngologists demonstrated most resident and specialist positions, but this dominance flipped once they reached the attending level which could potentially account for the lower number of female first authors seen in the top 50 rhinology papers assessed in this study [39]. This same pattern has been demonstrated in the field of otolaryngology in the United States as well, where women are underrepresented at all leadership levels [26]. To the best of our knowledge, there have been no published works that investigate the gender disparity in otolaryngology in England.

In conclusion, we analyzed the top 50 most frequently cited articles in rhinology of all time. The top 3 papers were a position paper on the classification and treatment of chronic rhinosinusitis with and without nasal polyps, a multicenter clinical study on olfactory and gustatory dysfunction relating to COVID-19, and a technique study on the vascular pedicle nasoseptal flap [20, 40, 41]. Overall, most articles had male first authors and male senior authors. There seems to be a trend towards increasing female authorship as there was a significant increase in female first authors after 2000; however, women remain underrepresented in rhinology paper authorship. The most common publication type was clinical research studies. The United States published the most articles but had the lowest number of female first authors compared to England. The trends seen in rhinology publications are a positive step forward to decrease gender disparities in academic medicine and to encourage continued efforts to reach equity and adequate representation.

There are several limitations to our study. Firstly, regarding the time-sensitive nature of the data collection, articles published near or after we performed our data collection would not have had time to accumulate many citations regardless of if they were the most impactful. Most of the articles in our study were from 1990 to 2010, which makes this issue less likely but still a consideration. As has been done in other studies, the only database utilized was the Web of Science, thus it is possible that other articles were not considered in our analysis. Articles were considered “top articles” solely based on the number of citations therefore it is possible that the determination of “top articles” was not completely comprehensive considering all aspects of the papers.

## Conclusion

This study identifies the most influential literature in the field of rhinology and highlights significant gender disparities in authorship. The most influential studies are consensus statements, large multicenter studies, and technique studies. Female otolaryngologists are underrepresented in the publication of rhinology-related clinical research, basic science studies, and review articles.

## Data Availability

Not applicable.
